# Deterministic control of magnetic vortex wall chirality by electric field

**DOI:** 10.1038/s41598-017-07944-9

**Published:** 2017-08-08

**Authors:** R. P. Beardsley, S. Bowe, D. E. Parkes, C. Reardon, K. W. Edmonds, B. L. Gallagher, S. A. Cavill, A. W. Rushforth

**Affiliations:** 1 School of Physics and Astronomy, University of Nottingham, Nottingham, NG7 2RD United Kingdom; 2 Diamond Light Source Chilton, Didcot, Oxfordshire OX11 0DE UK; 3 Department of Physics, University of York, Heslington, York, YO10 5DD United Kingdom

## Abstract

Concepts for information storage and logical processing based on magnetic domain walls have great potential for implementation in future information and communications technologies. To date, the need to apply power hungry magnetic fields or heat dissipating spin polarized currents to manipulate magnetic domain walls has limited the development of such technologies. The possibility of controlling magnetic domain walls using voltages offers an energy efficient route to overcome these limitations. Here we show that a voltage-induced uniaxial strain induces reversible deterministic switching of the chirality of a magnetic vortex wall. We discuss how this functionality will be applicable to schemes for information storage and logical processing, making a significant step towards the practical implementation of magnetic domain walls in energy efficient computing.

## Introduction

**Figure 1:**
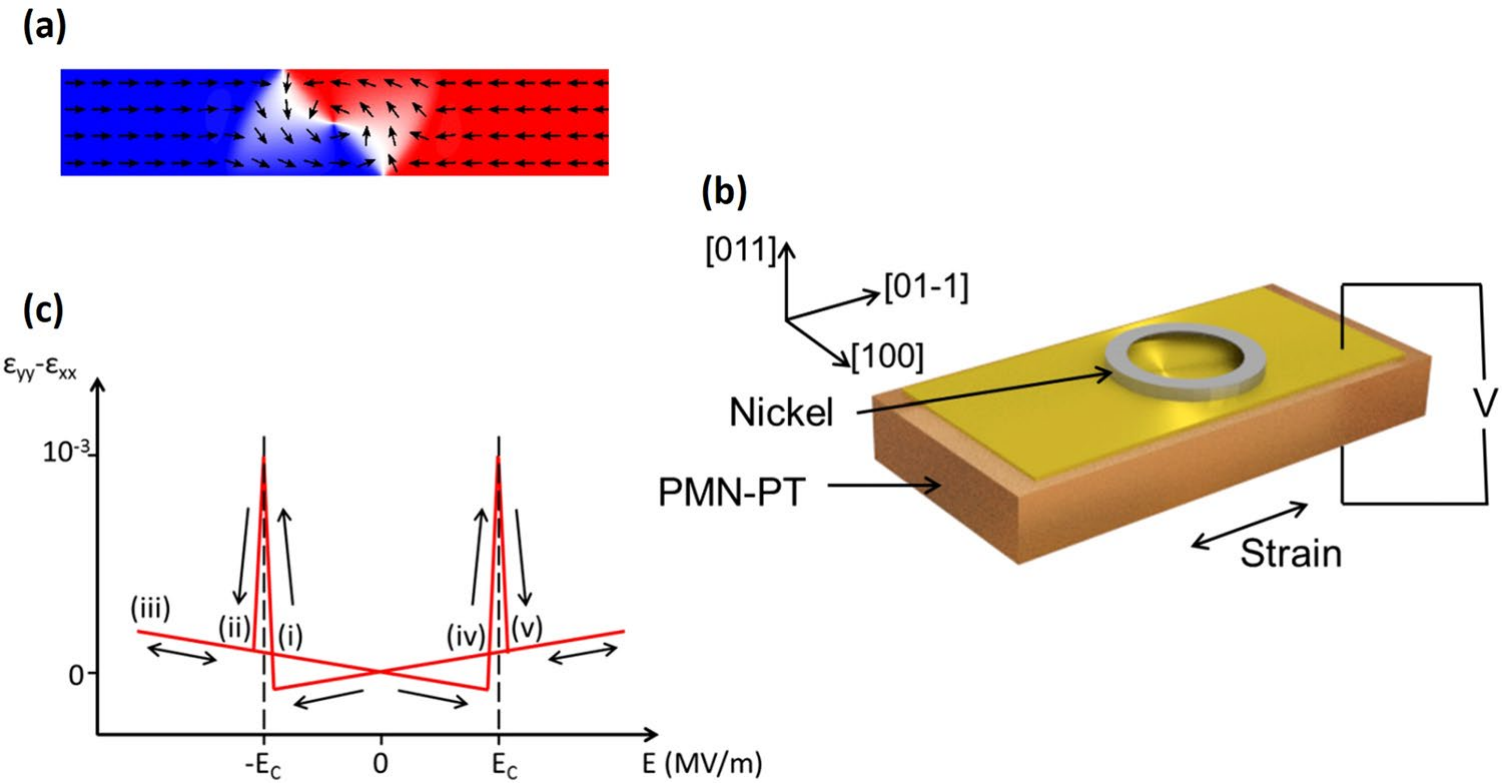
Device geometry and characteristics. (**a**) A representation of a vortex domain wall situated in a magnetic nanowire. Arrows represent the direction of the magnetisation. (**b**) A schematic of the device consisting of a nickel ring situated on top of a PMN-PT chip. A voltage applied across the thickness of the chip generates a uniaxial strain in the plane. (**c**) Schematic representation of the uniaxial strain as a function of the electric field applied to the PMN-PT, based on ref. 23. E_C_ is the ferroelectric coercive field. Roman numerals correspond to the electric field values labelled in Fig. 2.

Magnetic domain walls separate regions of a ferromagnetic material in which the magnetisation points in different directions. If the magnetic material is patterned into a quasi 1D object, such as a nanowire, the domain walls separate regions in which the magnetisation points in opposite directions and can be propagated along the length of the nanowire by the application of magnetic fields or electrical currents. This is highly attractive for implementation in magnetic data storage^1, 2^ and concepts for performing logical operations^3^ in which information is encoded as the presence or position of a domain wall. It has also been proposed to use the internal structure of the domain wall to encode information. For example, depending on the wire geometry and magnetic parameters, the domain wall structure can be that of a vortex consisting of a circulating pattern of magnetic moments in the plane of the wire, which point out of the plane at the centre of the wall (Fig. 1(a)). The sense of circulation of the magnetisation, i.e. the chirality of the wall, can be used to encode information as binary “1” or “0”^4^, and nanowire networks designed to use vortex domain walls to perform the key logical processing operations have been proposed^5^. In such schemes it is proposed to switch the chirality by driving the domain wall through a nanowire network containing notches via the application of an external magnetic field or electrical current. Experimentally, chirality rectification has been achieved by propagating vortex domain walls along wires with asymmetric notches^6^, rectangular end geometries^7^ and around curved sections of wires^8^ or rings^9^ using external magnetic fields. Similarly, the magnetic vortex states in nanodisks have been reversed by tailored magnetic field pulses^10, 11^. The use of externally applied magnetic fields creates limitations in terms of scalability and the ability to address individual domain walls, while the use of electrical currents causes Joule heating and produces stray magnetic (Oersted) fields which limit packing density due to interactions between neighbouring elements. In order to utilise vortex domain walls in any commercially viable technology it will be necessary to develop electrical methods to control the chirality of individually addressable domain walls reversibly and with low power.

Electric fields can provide a practical and energy efficient means to control magnetisation because their application does not require large current densities and does not generate Oersted magnetic fields. One route to achieve coupling of the electric field to the magnetisation is via mechanical strain in a hybrid piezoelectric/ferromagnet structure. A voltage applied to the piezoelectric component induces a mechanical deformation which transfers to the ferromagnetic layer and alters the magnetic anisotropy via inverse magnetostriction. The method has been used to modify magnetic anisotropy and to demonstrate rotation of the magnetic easy axis direction^12, 13^. Detailed modelling predicts that the implementation of these methods in devices consisting of single domain magnetic elements could lead to information storage devices with orders of magnitude better energy efficiency than present technologies relying on electrical currents^14^. To date, the use of inverse magnetostriction to control domain walls has resulted in movement between random pinning sites^15, 16^ or between positions along the circumference of a ring structure^17, 18^, local pinning of a domain wall at a position in a nanowire^19^, pinning at mobile ferroelectric domain walls^20^ and tuning of the domain wall velocity^21^ or width^22^. The possibility of switching the domain wall configuration by electric fields has not been considered before now.

In this article we demonstrate that the chirality of a vortex domain wall can be controlled reversibly by creating a strain-induced inhomogeneous uniaxial magnetic anisotropy energy profile in the vicinity of the domain wall. Using micromagnetic calculations we simulate the magnetisation dynamics in response to a strain pulse and show that the chirality switching can arise from the nucleation of a second vortex core followed by the damped precessional motion of the cores. We discuss how the method might be implemented in device concepts for low energy electric field operated non-volatile information storage and logical processing. The use of electric fields to manipulate the configuration of magnetic domain walls will reduce the energy required to switch the magnetic state in non-volatile information storage and processing technologies by orders of magnitude.

The experimental demonstration was achieved by positioning a vortex domain wall along the circumference of a ring structure, close to the axis of the uniaxial anisotropy. The device (Fig. 1(b)) consists of a piezoelectric [Pb(Mg_1/3_Nb_2/3_)O_3_]_0.68_ -[PbTiO_3_]_0.32_ (PMN-PT) (011) substrate with top and bottom electrodes, onto which is fabricated a 20 nm thick Ni ring of outer(inner) diameter 7.7μm (5.7 μm). Application of an electric field between the top and bottom electrodes results in mechanical deformation of the PMN-PT substrate and induces a uniaxial strain of order 10^−3^ in the plane of the Ni ring. Images of the magnetic contrast were obtained using the x-ray photoemission electron microscope (X-PEEM) on beamline I06 at the Diamond Light Source synchrotron.

**Figure 2:**
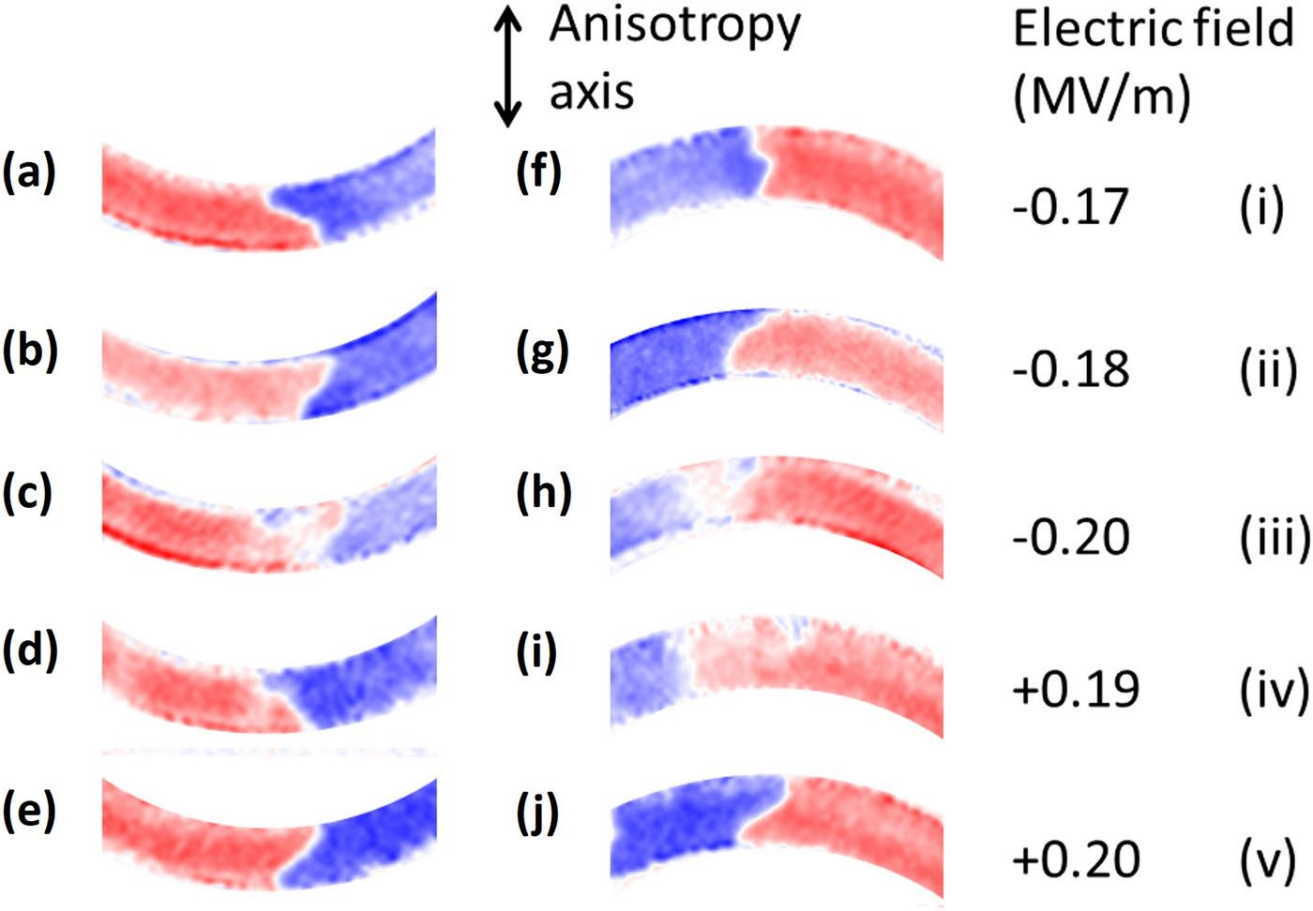
XMCD-PEEM images of strain-induced chirality switching in vortex domain walls. Red shading represents magnetisation pointing from right to left. Blue represents magnetisation pointing from left to right. Panel sequences (**a**) to (**e**) and (**f**) to (**j**) show the evolution of a tail to tail and head to head vortex wall respectively as a function of the electric field applied to the PMN-PT. The axis of the induced uniaxial anisotropy is represented by the arrow. Roman numerals correspond to the electric fields labelled in Fig. 1(c).

The application of an electric field across the thickness direction of a PMN-PT (011) substrate is known to result in a two stage reversal of the ferroelectric polarisation vector at the ferroelectric coercive field E_C_^23^. Switching of the polarisation vector between the <111> directions with components pointing out of the plane is mediated by a range of electric field for which the polarisation points in the plane. This is accompanied by a large uniaxial strain of order 10^−3^ in the plane in a narrow range of electric field around E_C_, which is approximately 0.18–0.19MV/m for our device (Fig. 1(c)). This results in a uniaxial anisotropy energy of order 10 kJm^−3^ ^12^. Further increase of the electric field results in the strain returning to a value close to that at zero field as the ferroelectric vector rotates out of the plane towards the < 111 > directions. The sequence of magnetic contrast images in Fig. 2 reveals that this transition is accompanied by a reversal of the vortex wall chirality. Figure 2(a) and (b) show the transition from a clockwise tail to tail wall to an anti-clockwise tail to tail wall at electric fields illustrated by points (i) and (ii) in Fig. 1(c). The same transition occurs between Fig. 2(d) and (e) for the opposite sign of applied electric field ((iv) and (v) in Fig. 1(c)), but an identical uniaxial strain transition. The intermediate magnetic state was not captured for these transitions because of the sharpness of the transition at E_C_ resulting in the uniaxial strain state being induced and removed between successive images. An intermediate magnetic state can be observed in Fig. 2(c) when the electric field had been increased further beyond the ferroelectric coercive field to point (iii) in Fig. 1(c). Here a reversible uniaxial strain is also present, albeit smaller than that generated at the ferroelectric coercive field. Removal of this uniaxial strain results in the switching of the vortex wall chirality, from anti-clockwise to clockwise in the sequence from Fig. 2(b) to (d). To summarise, Fig. 2(a) to (e) show that three successive applications of a uniaxial strain induce three successive reversals of the vortex wall chirality. The chirality switching is also observed for a head to head vortex wall shown in Fig. 2(f) to (j), but the sequence is interrupted by the movement of the domain wall along the circumference of the ring in Fig. 2(h) to (j). The importance of pinning of the domain wall position will be discussed in the next section.

A full understanding of the mechanism by which the vortex domain wall chirality switches would require knowledge of how the strain profile varies, both spatially and temporally. The structure of ferroelectric domains in PMN-PT and the processes involved in switching of the ferroelectric polarisation vector are areas of intense research at present. It is known that domains can range in size from tens^24^ or hundreds^12^ of nm up to several tens of µm^24^ and that different domain regions can represent different polarisation vectors as well as different structural phases^25^ because the [Pb(Mg_1/3_Nb_2/3_)O_3_]_0.68_ -[PbTiO_3_]_0.32_ composition sits close to a morphotropic phase boundary. The application of an electric field can induce reorientation of nanoscale domains and movement of the boundaries of micron scale domains within the same sample^24^. For our sample we cannot image the ferroelectric domain structure because the presence of the top electrode prevents escape of photoelectrons from the PMN-PT. We can, however consider the extreme cases: (i) the strain in the region of the vortex domain wall switches homogeneously (e.g. via the reorientation of nanoscale ferroelectric domains of size much smaller than the vortex domain wall) and (ii) the change in strain sweeps across the device (e.g. due to a ferroelectric domain wall moving across the region beneath the vortex domain wall). We find that both mechanisms can induce switching of the vortex wall chirality. We describe the first mechanism in detail here because the results of micromagnetic simulations most closely match the experimental results obtained, and the results of our investigations of the second mechanism are described in the Supplementary Note.

**Figure 3:**
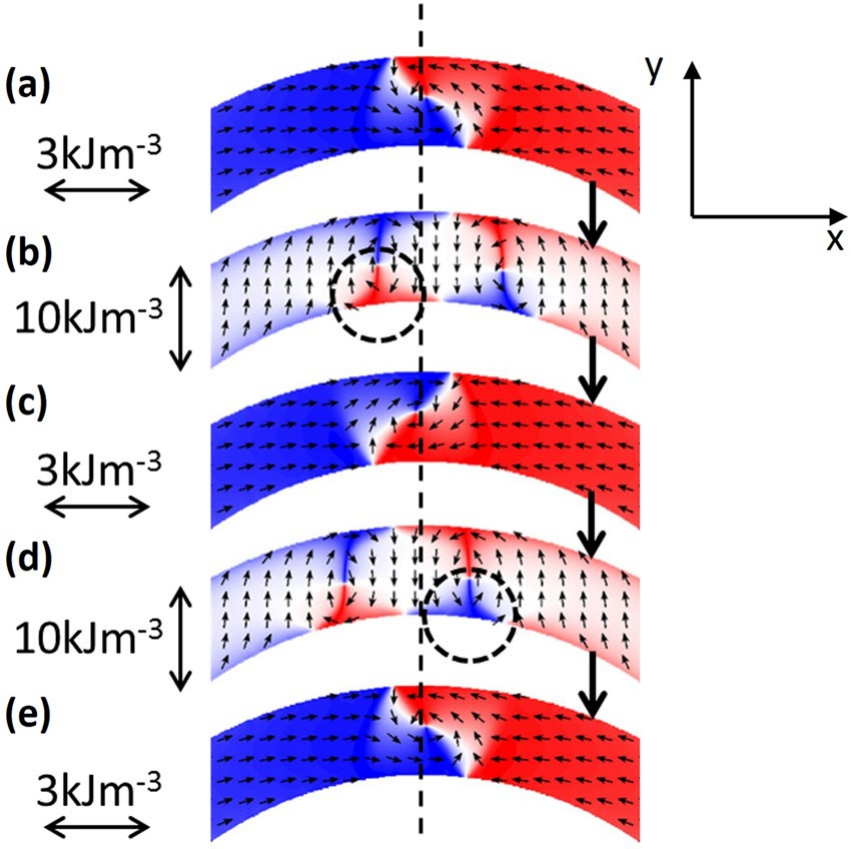
Micromagnetic simulations of a head to head vortex domain wall under the action of a uniaxial magnetic anisotropy energy. **(a**) to (**e**) show successive switching of the chirality of the vortex wall. The transitions from panels (a) and (c) to panels (c) and (e) respectively involve the reversal of the x-component of the magnetisation in the regions marked by dashed circles in (**b**) and (**d**).

We performed micromagnetic simulations using the OOMMF simulation package^26^ using realistic parameters for nickel (see Methods). Results from the simulations, presented in Fig. 3(a) to (e), show successive switching of vortex wall chirality from anticlockwise to clockwise and back to anticlockwise. The switching events are induced by the application of a uniaxial magnetic anisotropy favouring an easy axis perpendicular to the circumference of the ring in the vicinity of the domain wall, i.e. along the y-axis. The anisotropy causes a flux closure domain pattern to form, characterised by red and blue triangular regions in Fig. 3(b) and (d), in order to minimise the magnetic free energy. There are similarities between these patterns and the experimental image in Fig. 2(c). Crucially, the final position of the flux closure pattern in Fig. 3(b) and (d) is offset slightly to the right or left of the axis of the anisotropy depending upon the initial chirality of the domain wall. When the anisotropy is changed to an easy axis tangential to the ring in the region of the domain wall (i.e. parallel to the x-axis), the flux closure pattern evolves back to a vortex domain wall, with the opposite chirality to the original state.

We now examine in detail the important transitions involved in this process. In Fig. 3(a) the initial vortex domain wall state is made to resemble that in the experimental images, in particular the “s-shape” of the central white region, by applying a uniaxial anisotropy of K_U_ = −3 kJm^−3^ parallel to the x-axis. Here the minus sign denotes the axis of the anisotropy such that a positive or negative value for K_U_ represents an easy axis parallel to the y or x axis respectively. A small uniaxial anisotropy could arise during fabrication of the rings due to different thermal contraction of the Ni and PMN-PT, or due to the ferroelectric domain state prior to electric field poling^12^. Upon the application of a uniaxial anisotropy of K_U_ = +10 kJm^−3^ (easy axis along the y-axis, also represented by the dashed line in Fig. 3), the flux closure pattern forms by reversing the direction of magnetisation in a region on the inner circumference of the ring, indicated by the dashed circles in Fig. 3(b) and (d). The position of the reversed region depends upon the initial chirality of the domain wall, such that the flux closure pattern is made up of two vortex cores of opposite chirality – one from the original domain wall and one formed when the magnetisation in the region indicated by the dashed circle reverses. In our simulation, the reversal occurs by the precessional dynamics of the magnetisation, which is triggered by abruptly changing the anisotropy, and allowed to proceed using a realistic damping coefficient of α = 0.02. In the laboratory experiment, we do not know the timescale over which the anisotropy changes. This will likely be governed by the reorientation of ferroelectric domains or the movement of ferroelectric domain walls between pinning sites triggered by thermal activation. If the anisotropy changes on a timescale that is long compared to the damping of the magnetisation (a few ns), then the reversal of the magnetisation can still occur through thermal activation of the magnetisation precession. We have not investigated such processes. Once the region indicated has reversed, then the flux closure pattern oscillates along the circumference of the ring and settles to the final position on a timescale of several nanoseconds. The sequences in Supplementary Videos 1 and 2 show this oscillatory behaviour for the transitions in Fig. 3(a) to (b) and (c) to (d) respectively. The formation of the reversed region causes an initial rapid movement of the pattern to the left or right as the vortex cores gyrate. The direction of this initial motion is determined by the chirality of the original domain wall. The final offset of the flux closure pattern along the ring is determined by this initial motion of the vortices, after which damping of the magnetisation reduces subsequent oscillations. Upon setting the anisotropy back to K_U_ = −3 kJm^−3^ the formation of the vortex wall proceeds by domain wall motion resulting in the annihilation of the inner triangular region adjacent to the region marked by the dashed rings in Fig. 3(b) and (d). This sequence is shown in Supplementary Videos 3 and 4 for the transitions in Fig. 3(b) to (c) and (d) to (e) respectively. For these transitions to the single domain wall states the damping parameter was set to a large value (α = 0.5) to suppress motion of the domain wall around the ring. If a more realistic value for the damping parameter is used (α = 0.02) then the domain wall moves around the ring by 90°. Experimentally, we do not observe such motion due to pinning of the domain wall, likely by material defects or edge roughness. The exception to this is the sequence in Fig. 2(h) and (i). Previous studies^17, 18^ of vortex domain walls in Ni-ring/PMN-PT devices observed rotation of the domain wall position around the ring, but did not report switching of the chirality.

The sequences in the simulations are deterministic. The initial wall chirality determines the offset of the flux closure pattern which in turn determines the chirality of the newly formed domain wall. In real devices the final position of the flux closure pattern will depend on several factors including the ring dimensions, defects, edge roughness, the size of the induced strain, thermal fluctuations and material parameters such as the magnetisation and damping. Such factors may introduce some stochasticity into the magnetisation dynamics which could prevent repeatable switching of the chirality. A detailed investigation of the influence of such factors is beyond the scope of the present work. It should be possible however, to set the chirality of the domain wall by setting the offset of the flux closure pattern using an external impetus, such as a weak magnetic field pulse, or an electrical current pulse.

**Figure 4:**
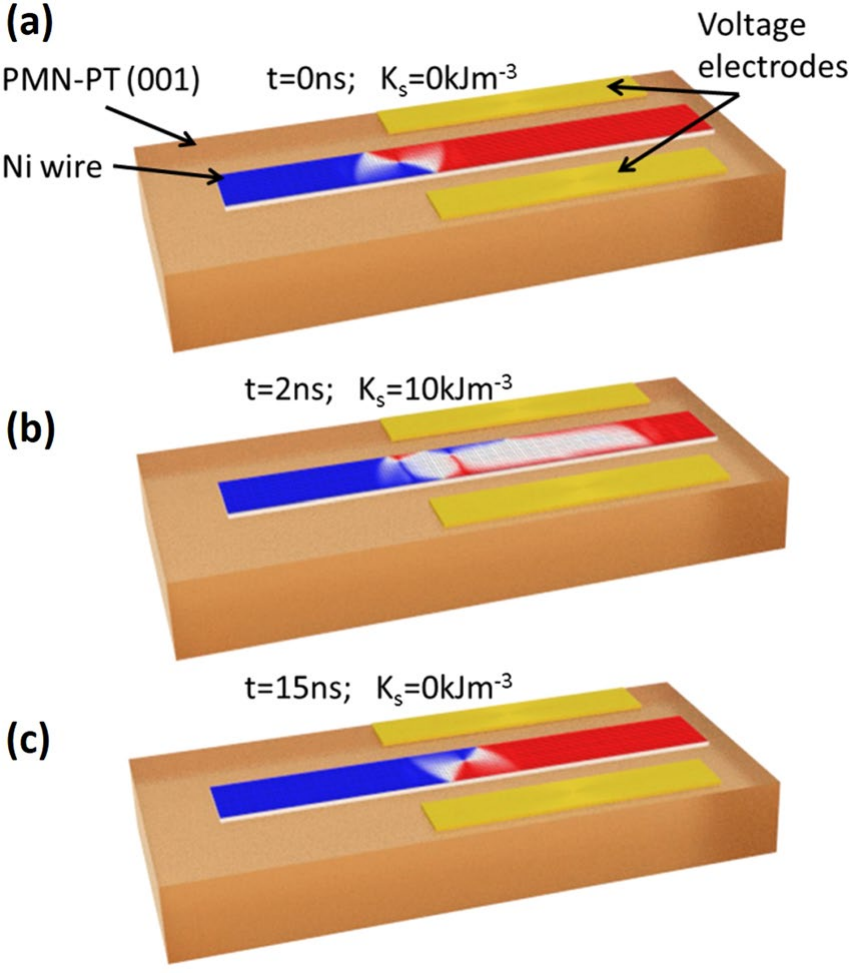
A method to switch the chirality of a vortex domain wall by applying strain locally. A schematic representation of a device element for reversing the chirality of a vortex domain wall in a nanowire fabricated on a piezoelectric substrate. A voltage applied to the electrodes induces a mechanical strain and a uniaxial anisotropy favouring an easy axis transverse to the wire. (**a**), A vortex domain wall positioned at the edge of the electrode region is transformed to a flux closure domain pattern near the electrodes (**b**). (**c**), Relaxation of the induced anisotropy leads to the formation of a vortex domain wall with the opposite chirality to the initial domain wall.

The functionality described here has potential for application in devices for information storage and logical processing. The inhomogeneous magnetic anisotropy profile required to switch the chirality of a vortex domain wall could also be induced in a straight nanowire with voltage gate electrodes positioned to produce a strain in a local region along the wire. The geometry is shown in Fig. 4 and simulations of the chirality reversal in response to a voltage pulse are presented in Supplementary Video 5. The wire could form part of a larger nanowire circuit used for logical processing based on domain walls. A gate design that produces an anisotropy gradient may also be used to move the domain wall along the wire. Although the investigation of domain wall motion in magnetic anisotropy gradients is beyond the scope of the present work, we suggest that a device design in which a succession of gates are implemented to move and switch the chirality of vortex domain walls would avoid the need to use magnetic fields or electrical currents in information processing schemes using domain walls, thereby removing some of the major practical limitations to the development of such technologies.

## Methods

10 mm × 10 mm × 0.5 mm PMN-PT(110) substrates were purchased from Atom Optics Co., Ltd. Atomic Force Microscopy measurements revealed a surface roughness of order 1 nm. Ti(5 nm)/Au(35 nm) electrodes were deposited onto the top and bottom sides of the PMN-PT by thermal evaporation. The ring pattern was defined using electron beam lithography before deposition of Ni(20 nm)/Al(2 nm) by magnetron sputtering followed by lift-off.

Magnetic contrast images were obtained using the x-ray photoemission electron microscope (PEEM) on beamline I06 at the Diamond Light Source synchrotron. Illuminating the sample at oblique (16°) incidence and making use of XMCD at the Ni L_3_ edge (853 eV) as the contrast mechanism allowed sensitivity to in-plane moments with a spatial resolution of approximately 50 nm. Azimuthal rotation of the sample with respect to the incident polarization vector allowed unambiguous assignment of the magnetization direction in each domain. Additional electrical feedthroughs allowed the *in-situ* application of voltage to the PMN-PT whilst imaging.

Micromagnetic simulations were carried out using the Object Oriented Micromagnetic Framework (OOMMF) package^26^ installed on the University of Nottingham High Performance Computing cluster. Simulations were performed for a half ring geometry with inner diameter = 5.7 µm, outer diameter = 7.7 µm and thickness = 20 nm. A zoomed-section is shown in Fig. 3. The mesh size was 5 nm × 5 nm × 20 nm. Magnetisation M = 490 kAm^−1^. The damping constant is stated in the main text. The ends of the half ring section were set to critical damping to suppress the reflection of spin waves. Simulations were carried out using uniaxial anisotropy energy values of K_U_ = −3 kJm^−3^ and +10 kJm^−3^.

## Electronic supplementary material

Supplementary Information

Supplementary Video 1

Supplementary Video 2

Supplementary Video 3

Supplementary Video 4

Supplementary Video 5

Supplementary Video 6
