# Supplementary material for: Deterministic control of magnetic vortex wall chirality by electric field
Source: Sci Rep. 2017 Aug 8;7:7613. doi: 10.1038/s41598-017-07944-9 (PMC5548751; doi:10.1038/s41598-017-07944-9)
Supplement: Supplementary file 1 — Supplementary Information [file 41598_2017_7944_MOESM1_ESM.pdf]

Deterministic control of magnetic vortex wall chirality by electric field

Supplementary Note

R. P. Beardsley, S. Bowe, D. E. Parkes, C. Reardon, K. W. Edmonds, B. L. Gallagher, S. A. Cavill*

and A. W. Rushforth*

*Corresponding authors: andrew.rushforth@nottingham.ac.uk and stuart.cavill@york.ac.uk

Magnetic vortex domain wall chirality switching induced by a slowly sweeping strain profile

The micromagnetic simulations presented in the main text consider the case where a homogeneous uniaxial strain is applied on timescales short compared to the magnetisation dynamics. It is postulated that the same results would be obtained if a homogeneous strain profile was applied on timescales longer than the magnetisation dynamics if thermal activation was responsible for triggering switching of the magnetisation in certain regions of the device. Here we consider an alternative scenario in which the uniaxial strain is swept from one side of the ring structure to the other on timescales long compared to the magnetisation dynamics. Micromagnetic simulations were performed using OOMMF with the same parameters as described in the main text. The damping parameter was set at α=0.02 to simulate realistic damping. A uniaxial anisotropy energy of magnitude K_U=10kJm^{-3} was applied to one region of the structure, the size of which was increased in 0.1µm increments with the magnetisation dynamics allowed to damp fully at each increment. This would represent a slowly creeping strain profile, such as might occur due to the motion of a ferroelectric domain wall on timescales long compared to the magnetisation dynamics. The sequences in Supplementary Fig. S1(a) show the results of simulations as the uniaxial anisotropy is swept from left to right. As the boundary between the regions with different anisotropy approaches the vortex domain wall, the wall suddenly moves towards the boundary and is absorbed by the high anisotropy region, reversing its chirality in the process. This part of the sequence is shown in Supplementary Video 6. The switching of the chirality occurs because the magnetisation on the left

hand side of the vortex domain wall has a component along the y-axis that is antiparallel to the corresponding component of magnetisation *within* the left hand side of the wall. This situation is energetically unfavourable and the total energy of the system is reduced by the switching of the vortex wall chirality. Consistent with this interpretation are the results of sweeping the anisotropy from right to left, as shown in Supplementary Fig. S1(b). The magnetisation on the right hand side of the vortex domain wall has a component along the y-axis that is parallel to the magnetisation within the wall. This situation is energetically favourable and so the chirality of the domain wall does not switch.

Supplementary Figs. S2(a) and (b) show the results of reducing the anisotropy. Starting with the final state from Supplementary Fig. S1(a), the anisotropy is reduced to K_U=-3kJm^{-3} (i.e. an easy axis parallel to the x-axis) in regions spreading from right to left (Fig. S2(a)) or from left to right (Fig. S2(b)). In Fig. S2(a) the chirality of the vortex domain wall is retained, where as in Fig. S2(b) the chirality switches once again. In both cases the vortex domain wall moves with the boundary separating the regions with different anisotropy.

The sequences in Supplementary Figs. S1 and S2 do not resemble the important features in the experimentally obtained images in Fig. 2, while the results of simulations involving homogeneous strain-induced anisotropy in Fig. 3 do resemble the experimental images. This leads us to conclude that in the experimental device the strain was induced homogeneously, or that if the strain swept across the region of the vortex domain wall, it did so on timescales much faster than the magnetisation dynamics. The results of the simulations presented in this section do reveal alternative mechanisms by which spatially and temporally varying strain profiles can be used to control both the chirality and position of a vortex domain wall. Sweeping the strain across the device along other directions, for example from top to bottom, may provide interesting avenues for further study.

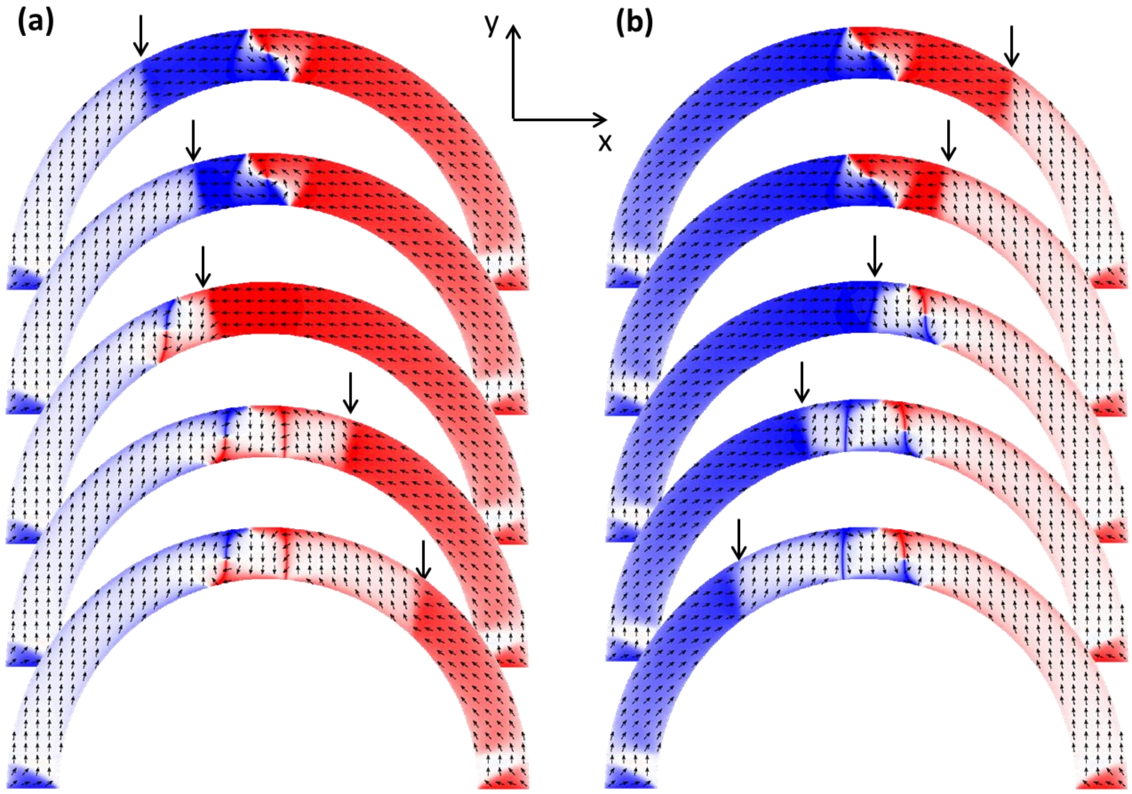

Supplementary Figure S1. Sweeping a uniaxial anisotropy across the device. Micromagnetic simulations of a vortex domain wall under the action of a uniaxial magnetic anisotropy energy that is swept **(a)** from left to right, and **(b)** from right to left. Arrows show the boundary between the regions of high and low anisotropy energy.

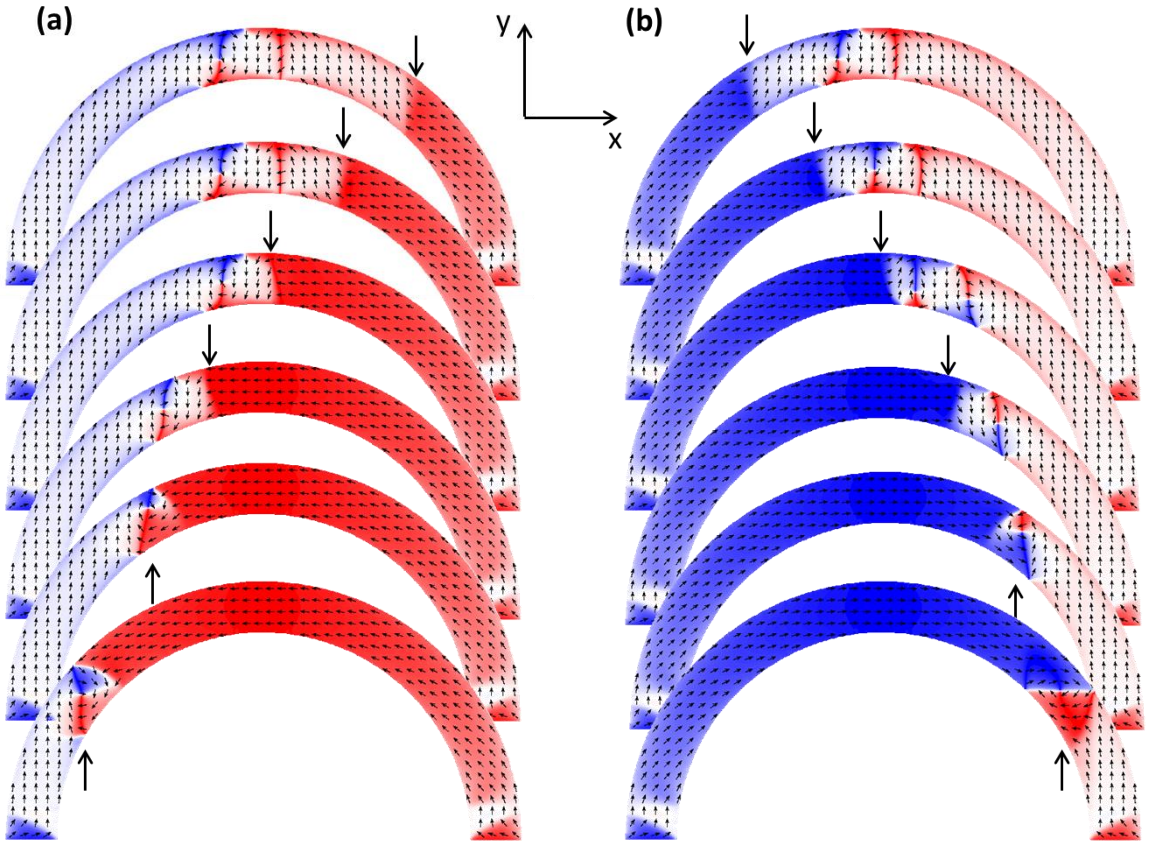

Supplementary Figure S2. Relaxing the uniaxial anisotropy across the device. Micromagnetic simulations starting with final states from Supplementary Fig. S1(a) and reducing the uniaxial anisotropy **(a)** from right to left and **(b)** from left to right. Arrows show the boundary between the regions of high and low anisotropy energy.

Supplementary Video 1. Oscillating vortex cores. The oscillation of the vortex cores around the circumference of the ring during the transition from Fig. 3(a) to (b).

Supplementary Video 2. Oscillating vortex cores. The oscillation of the vortex cores around the circumference of the ring during the transition from Fig. 3(c) to (d).

Supplementary Video 3. Reforming the vortex wall. The formation of the vortex domain wall during the transition from Fig. 3(b) to (c).

Supplementary Video 4. Reforming the vortex wall. The formation of the vortex domain wall during the transition from Fig. 3(d) to (e).

Supplementary Video 5. Switching the vortex domain wall chirality in a nanowire. Shows the time resolved dynamics of a vortex domain wall in a nanowire when a uniaxial anisotropy is induced in part of the nanowire, then subsequently removed.

Supplementary Video 6. Switching the vortex domain wall chirality in a nanowire. Shows the switching of the vortex wall chirality during the sequence in Fig. S1(a).
